# Efficacy of dual triggering in poor ovarian responders defined according to Bologna and POSEIDON criteria: a systematic review with meta-analysis

**DOI:** 10.1007/s10815-026-03821-5

**Published:** 2026-02-06

**Authors:** Antonio Mercorio, Alessandro Conforti, Nicola Pluchino, Panagiotis Drakopoulos, Matteo Giudice, Pierluigi Giampaolino, Alexandre Vallee, Vehbi Yavuz Tokgoz, Carlo Alviggi, Jean Marc Ayoubi

**Affiliations:** 1 Assisted Reproductive Technology Unit, Department of Gynecology, Hôpital Foch, Suresnes, France; 2 Department of Public Health, University of Naples Federico II, Naples, Italy; 3 Department of Neurosciences, University of Naples Federico II, Naples, Italy; 4 Division of Gynecology, Department Woman-Mother-Child, Centre Hospitalier Universitaire Vaudois (CHUV), University Hospital of Lausanne, Lausanne, Switzerland; 5 Institute of Life, IVF Unit, Athens, Greece; 6 Faculty of Medicine, European University Cyprus, Nicosia, Cyprus; 7 Centre for Reproductive Medicine, Universitair Ziekenhuis Brussel, Vrije Universiteit Brussel, Brussels, Belgium; 8 Department of Public Health, University of Naples Federico II, Naples, Italy; 9 Department of Epidemiology-Data-Biostatistics, Delegation of Clinical Research and Innovation (DRCI), Foch Hospital, Suresnes, 92150 France; 10 Reproductive Endocrinology and Infertility Unit, Department of Obstetrics and Gynecology, School of Medicine, Eskişehir Osmangazi University, Eskişehir, Turkey

**Keywords:** Dual triggering, Ovulation induction, IVF, ART, Ovarian stimulation, Poor ovarian responders

## Abstract

**Purpose:**

To compare dual triggering versus hCG-only triggering in poor responders undergoing controlled ovarian stimulation with a GnRH antagonist protocol in ART cycles.

**Methods:**

A systematic review and meta-analysis was performed. The primary outcome was the number of mature oocytes (MII). The number of oocytes retrieved, clinical pregnancy, and miscarriage rates were analyzed as secondary outcomes. Subgroup analyses were conducted according to the Bologna and POSEIDON classifications.

**Results:**

Ten studies were included. Dual triggering significantly increased the number of retrieved and mature oocytes in patients classified according to the Bologna criteria, but not in those classified by the POSEIDON criteria. Age-related differences across studies appeared to influence the efficacy of dual triggering. A borderline improvement in clinical pregnancy rates was observed among Bologna-defined patients. Miscarriage rates did not differ significantly between the groups.

**Conclusions:**

Dual triggering appears to improve oocyte yield and maturity in Bologna-defined patients, but this effect is unlikely to apply uniformly across all phenotypes. Persistent heterogeneity in poor responder definitions limits the understanding of the benefits of dual triggering in this population. Well-designed prospective studies with rigorous phenotypic stratification are warranted to identify which patients are most likely to benefit from dual triggering.

**Supplementary Information:**

The online version contains supplementary material available at 10.1007/s10815-026-03821-5.

## Introduction

Poor ovarian responders (PORs) represent a substantial proportion of the assisted reproductive technology (ART) population and pose a significant clinical challenge. Due to a deficiency in both oocyte reserve and competence, in vitro fertilization (IVF) outcomes in these patients remain poor.

Oocyte maturation triggering is a critical step in IVF. For decades, hCG has been used as a single agent to induce final oocyte maturation, given its structural and functional similarity to LH [1]. However, among high responders, growing evidence has revealed that dual triggering with GnRHa, by allowing a reduced hCG dose, decreases the risk of OHSS [2], while in normal responders, it has been shown to improve oocyte yield and maturation via the flare-up effect that mimics the natural mid-cycle FSH and LH surge and optimizes follicular response [3].

Consequently, dual trigger protocols are increasingly applied, with reported improvements in reproductive outcomes [3, 4].

To date, data on dual trigger in PORs remain limited and conflicting [5, 6].

The inconsistency of the results could be attributed, at least in part, to the lack of a well-established definition of poor responders and, consequently, to the heterogenicity of the examined population.

Polyzos et al. reported the use of as many as 41 distinct POR definitions in a total of 47 RCTs [7].

In an attempt to standardize the definition of POR, in 2011 the European Society of Human Reproduction and Embryology (ESHRE) proposed the Bologna criteria [8], requiring at least two of the following: advanced maternal age (≥ 40 years) or risk factors for POR, ≤ 3 oocytes retrieved after standard stimulation, or abnormal ovarian reserve tests (AFC < 7 or AMH 0.7–1.3 ng/mL).

The validity of these criteria has been questioned later [9] and the POSEIDON (Patient-Oriented Strategies Encompassing IndividualizeD Oocyte Number) classification was proposed for a better categorization [10], which distinguishes expected (groups 3 and 4) from unexpected poor responders (groups 1 and 2) based on age, ovarian biomarkers, and previous stimulation outcomes.

The effect of dual triggering in poor responders remains uncertain, with the only available meta-analysis based on outcomes such as clinical pregnancy rate, implantation rate, and live birth rate [11]. These outcomes are prone to bias due to confounding factors such as male factor infertility and maternal characteristics not directly related to ovarian function or oocyte quality.

This study aims to clarify this issue by distinguishing, for the first time, between women classified according to the Bologna or POSEIDON criteria, with a specific focus on the number and maturity of retrieved oocytes—both strong and independent predictors of reproductive success in ART.

To investigate the efficacy of dual trigger versus hCG-only trigger in antagonist cycles among poor ovarian responders, we designed the present study based on a selected population defined according to the Bologna or POSEIDON criteria.

## Methods

### Search strategy for the identification of studies

No approval from the Institutional Research Ethics Committee was sought because of the nature of this work. The study is reported in accordance with the Preferred Reporting Items for Systematic Reviews and Meta-Analyses (PRISMA) 2020 guidelines [12].

A systematic literature search was performed in the PubMed/MEDLINE, Embase, and Scopus databases from January 2011 (the year of the Bologna criteria proposal) to February 20, 2025. For each database, the core concepts included: (1) trigger strategy (“dual trigger”, “double trigger”, “GnRH agonist trigger”, “hCG trigger”),(2) IVF/ICSI treatment, and (3) poor responder definitions (“ Bologna criteria”, “POSEIDON criteria”).

Filters were applied to restrict results to human studies and original research articles. Detailed examples of database-specific syntax are included: PubMed: (“dual trigger” OR “double trigger”) AND (“IVF” OR “ICSI”) AND (“poor ovarian responder*” OR “ Bologna criteria” OR “POSEIDON criteria”). Embase: analogous structure using Emtree terms and free-text fields. Scopus: TITLE-ABS-KEY search using the same conceptual combination.

In addition, the reference lists of relevant articles and reviews were manually screened to identify additional eligible studies.

### Study selection

Studies were selected according to the Population, Intervention, Comparison, Outcomes (PICO) model. The population included women undergoing IVF with poor ovarian response, defined according to the Bologna or the POSEIDON criteria. The intervention used dual triggering (GnRH agonist + hCG), compared with conventional hCG-only triggering. Studies reporting outcomes of interest were included regardless of study design (RCTs and retrospective studies). Only English-language, peer-reviewed full-text articles were included. Case reports, conference abstracts, gray literature, and duplicate publications were excluded.

A PRISMA flow diagram was constructed to illustrate the process of study identification, screening, eligibility assessment, and inclusion.

### Study outcomes

The primary outcome was the number of metaphase II (MII) oocytes, defined as the number of oocytes reaching the metaphase II stage. Secondary outcomes included the number of oocytes retrieved, defined as the total number of oocytes aspirated per oocyte retrieval procedure; the clinical pregnancy rate, defined as the ultrasonographic visualization of at least one gestational sac or the presence of definitive clinical signs of pregnancy; and the miscarriage rate, defined as the spontaneous loss of an intrauterine pregnancy before 22 completed weeks of gestation. All outcomes were determined in accordance with the International Glossary on Infertility and Fertility Care [13].

### Data extraction

Two independent reviewers (AN and AC) screened titles and abstracts for eligibility. Full-text articles were then reviewed for inclusion. Duplicates were removed using EndNote online software and manual checking. Data extraction was performed independently by two reviewers (AM and MG) using a standardized form based on the Cochrane Handbook template. Discrepancies were resolved through discussion with senior authors (NP, JMA).

For studies reporting medians with ranges instead of means and standard deviations, we estimated means and SDs using the method proposed by Hozo et al. [14].

### Risk of bias

The risk of bias in included studies was independently assessed by two reviewers (AM and AC), with discrepancies resolved by consensus with a third senior reviewer (CA).

For randomized controlled trials, the Cochrane Risk of Bias 2.0 (ROB 2.0) tool was used. For nonrandomized studies, the ROBINS-I tool (Risk Of Bias In Non-randomized Studies—of Interventions) was applied.

Publication bias was evaluated for the primary outcome (number of MII oocytes) by the combination of qualitative visual inspection of funnel plots and quantitative statistical test (Egger/Begg’s test) [15].

### Sensitivity analysis

A sensitivity analysis was performed for the primary endpoint (number of MII oocytes) excluding studies judged to be at risk of bias.

## Results

### Study selection

**Fig. 1:**
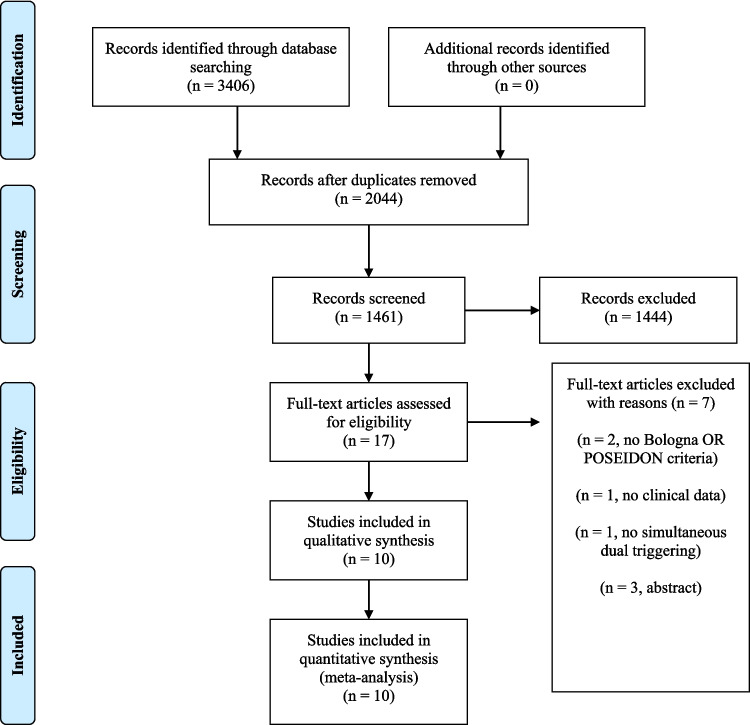
Study selection according to the PRISMA 2020 guidelines

A total of 3406 articles were identified through the search. Duplicates were removed manually and using the EndNote library (*n* = 1945). The titles and abstracts of 2044 papers were scrutinized, and 17 full-text articles were assessed for eligibility. Seven papers were excluded because they did not fulfil the inclusion criteria. A total of 10 articles were included in the qualitative and quantitative assessment (Fig. 1).

### Study design, year of publication, and country

This systematic review includes three randomized controlled trials (RCTs) and seven retrospective studies. The articles were published between 2016 and 2023 and were conducted in the following countries: Taiwan, Spain, Iran, Turkey, Egypt, and Brazil. The characteristics of the included studies are summarized in Table 1.

**Table 1:** Summary of studies evaluating dual trigger versus hCG-only trigger in poor ovarian responders

Author	Population	Study design	Participants Total (I/R)	Age (mean ± SD)	AMH	AFC	No. of oocytes retrieved (I vs R)	*p*-value	MII oocytes (I vs R)	*p*-value
Maged et al. [20] Egypt	Bologna criteria	RCT	160 (80/80)	39.1(I) vs 38.9 (R)	0.9 ± 0.1(R), 0.9 ± 0.1(I)	(R)4.5 ± 1.1 (I) 4.6 ± 0.9	5.3 ± 1.9 (I) vs 4.5 ± 2.4 (R)	0.014	3.8 ± 1.4 (I) vs 3.1 ± 1.7 (R)	0.004
Keskin et al. [16] Turkey	Poseidon 3/4	RCT	112 (57/55)	36.75 (I) vs 33.91 (R)	0.53 ± 0.34(R), 0.47 ± 0.29(I)	NA	5.23 ± 3.16 (I) vs 5.62 ± 4.27 (R)	0.974	3.58 ± 2.40 (I) vs 4.15 ± 3.21 (R)	0.559
Mutlu et al. [21]	Bologna criteria	Retrospective	1283(674/609)	36.7 (I) vs37.0 (R)	NA	(R)4.4 ± 1.7 (I) 4.4 ± 1.4	4.5 ± 2.4 (I)vs 3.1 ± 2.3 (R)	0.001	3.4 ± 2.0 (I) vs2.3 ± 2.9 (R)	0.001
Tulek et al. [17] Turkey	Poseidon 3	Retrospective	952 (315/637)	31.22 (I) vs 31.21 (R)	NA	NA	4.81 ± 1.74 (I) vs 4.20 ± 1.72 (R)	< 0.001	3.78 ± 1.55 (I) vs 2.85 ± 1.28 (R)	0.001
Tulek et al. [17] Turkey	Poseidon 4	Retrospective	2047 (753/1294)	39.66 (I) vs 39.60 (R)	NA	NA	3.83 ± 1.86 (I) vs 3.5 ± 1.49 (R)	< 0.001	3.09 ± 1.68 (I) vs 2.52 ± 1.21 (R)	< 0.001
Eser et al. [22] Turkey	Bologna criteria	Retrospective	109 (47/62)	35.3 (I) vs 35.8 (R)	NA	(R)4.7 ± 1.7 (I) 5.0 ± 1.8	2.2 ± 1.6 (I) vs 2.6 ± 1.8 (R)	0.227	0.8 ± 0.3 (I) vs 0.8 ± 0.3 (R)	0.834
Chern et al. [18] Taiwan	Poseidon 4	Retrospective	308 (194/114)	40.0 (I) vs 40.0 (R)	0.47 ± 0.31(R), 0.48 ± 0.26(I)	(R)2.5 ± 1.7 (I) 2.4 ± 1.3	3.3 ± 2.7 (I) vs 1.6 ± 1.5 (R)	< 0.001	2.6 ± 2.0 (I) vs 1.3 ± 1.0 (R)	0.001
Zhang et al. [23] (5000 UI) China	Bologna criteria	Retrospective	714 (386/328)	36.31 (I)vs35.94 (R)	0.48 ± 0.29 (R),0.47 ± 0.29(I)	(R)4.20 ± 1.32 (I). 4.26 ± 1.18	3 (0–11) (I) vs 2 (0–9) (R)	0.001	3 (0–10) (I)vs2 (0–8) (R)	0.001
Zhang et al. [23] (10,000 UI) China	Bologna criteria	Retrospective	675 (312/363)	36.39 (I)vs36.04 (R)	0.48 ± 0.28(R), 0.48 ± 0.30(I)	(R). 4.18 ± 1.26 (I) 4.26 ± 1.35	3 (0–11) (I) vs 2 (0–9) (R)	0.001	3 (0–9) (I)vs 2 (0–8) (R)	0.001
Eftekhar et al. [24] Iran	Bologna criteria	RCT	80 (41/39)	32.7 (I) vs 33.5 (R)	0.75 ± 0.48 R) 0.93 ± 0.52(I)	(R)5.0 ± 1.5 (I) 4.7 ± 1.8	4.5 ± 1.7 (I) vs 4.6 ± 1.7 (R)	0.89	3.9 ± 1.7 (I) vs 4.0 ± 1.8 (R)	0.80
De Oliveira et al. [25] Brasil	Bologna criteria	Retrospective	40 (18/22)	NA	NA	NA	7.3 ± 3.12 (I) vs 4.67 ± 1.63 (R)	< 0.005	5.38 ± 2.82 (I) vs 3.32 ± 1.24 (R)	< 0.005
Ozer et al. [19] Turkey	Poseidon 3	Retrospective	386 (105/281)	32.85 (I)vs32.2 (R)	0.353 (R) 0.55 ± 0.31 (I)	NA	NA	NA	2.62 ± 1.14 (I) vs 2.81 ± 1.18 (R)	0.143

### Population, size, age

Overall, this study included a total of 6,776 women: 2,982 in the dual trigger group and 3,794 in the hCG-only group (controls), all of whom presented a low response to conventional fertility treatment.

Four studies evaluated poor responders according to the POSEIDON criteria, including groups 3 and 4 [16–19] while seven studies classified patients according to the Bologna criteria [20–26].

Chern et al. [18] reported the oldest study population, with a mean age of 40 years, whereas Tulek et al. [15] included the youngest cohort, with a mean age of 31 years. The mean age across the other studies ranged from 32 to 39 years. Oliveira et al. [25] reported only that participants were younger than 40 years, without specifying a precise mean age.

### Controlled ovarian stimulation and trigger regimens

All studies included in the analysis used a GnRH antagonist protocol for pituitary downregulation.

For double triggering, 0.2 mg of triptorelin was used as the GnRH agonist in all studies except for Zhang et al. [23], who administered 0.1 mg of triptorelin, and Chen et al. [26], who used 2 mg of leuprolide.

Regarding the hCG trigger, most studies employed either 250 µg of recombinant hCG or 6500 IU of urinary hCG. Maged et al. [20] used the highest dose (10.000 IU), while Zhang et al. [23] administered 5000 IU in one subgroup and 10.000 IU in another (Table 2).

**Table 2:** Ovarian stimulation, triggering and luteal phase management

Author	Total gonadotropin dose used (IU)	Duration of stimulation (days)	Reference trigger	Intervention	Timing of triggering	Fresh embryo transfer (ET)/Frozen-thawed embryo transfer (FET)	Type and route of administration of progesterone for luteal phase support	Estradiol supplementation
Maged et al. [20] Egypt	(R) 4473 ± 652 (I). 4398 ± 661	(R). 13.1 ± 1.0 (I)13.4 ± 1.2	10.000 UI of urinary hCG	10.000 UI of urinary hCG + 0.2 mg of triptorelin	At least 3 follicles larger than 14 mm and one of them with a mean diameter of 17 mm or more	ET, day-3; number of embryos not specified	400 mg of natural progesterone twice daily intravaginal until the day of serum b-hCG assessment	No
Keskin et al. [16] Turkey	(R)3300 (1200–5400) (I) 3375 (900–6250)	(R) 9.95 ± 1.91 (I). 9.72 ± 2.38	250 mcg of recombinant hCG	250 mcg of recombinant hCG + 0.2 mg triptorelin acetate	When the leading follicle reached ≥ 17 mm in diameter	ET, day-3 or day-5; SET/DET according to age	600 mg of micronized progesterone daily intravaginal until the day of serum b-hCG assessment	No
Mutlu et al. [21]	(R)3072.3 ± 1076.1 (I) 3134.2 ± 767.4	(R) 10 (9–12) (I). 10 (9–11)	250 mcg of recombinant hCG	250 mcg of recombinant hCG + 0.2 mg triptorelin	When at least two follicles reached ≥ 17 mm in diameter	ET, day-3 or day-5; SET or DET	NA	NA
Tulek et al. [17] Turkey (POSEIDON 3)	(R)3511.62 ± 1049.29 (I) 3558.65 ± 1228.28	(R)9.52 ± 1.49 (I) 9.47 ± 1.48	250 mcg of recombinant hCG	250 mcg of recombinant hCG + 0.2 mg triptorelin acetate	At least one follicle reached a diameter of 18 mm	ET, day-3 or day-5; SET or DET	100 mg oral progesterone three times daily or 200 mg of micronized progesterone three times daily intravaginal until the 8th-10th gestational week	No
Tulek et al. [17] Turkey (POSEIDON 4)	(R)3385.10 ± 1032.11 (I) 3464.81 ± 943.87	(R)9.51 ± 1.55 (I) 9.42 ± 1.57	250 mcg of recombinant hCG	250 mcg of recombinant hCG + 0.2 mg triptorelin acetate	At least one follicle reached a diameter of 18 mm	ET, day-3 or day-5; SET or DET	100 mg oral progesterone three times daily or 200 mg of micronized progesterone three times daily intravaginal until the 8th-10th gestational week	No
Eser et al. [22] Turkey	(R)3839.5 ± 805.5 (I) 3165.4 ± 1124.2	(R) 8.5 ± 1.7 (I) 8.5 ± 2.1	250 mcg of recombinant hCG	250 mcg of recombinant hCG + 0.2 mg triptorelin acetate	When follicles reached ≥ 17 mm in diameter	ET, day-3, number of embryos not specified	90 mg of vaginal progesterone gel daily until the negative pregnancy test or 10 weeks of gestation	No
Chern et al. [18] Taiwan	(R1). 1561.4 ± 676.6 (I1). 1751.4 ± 708.9 *p value* 0.316 (R2). 2729.7 ± 1109.0 (I2). 2931.5 ± 779.9	(R)10.4 ± 2.7 (I) 10.6 ± 2.2	250 mcg of recombinant hCG	250 mcg of recombinant hCG + 2 mg of leuprorelin acetate	At least one follicle reached a diameter of 18 mm	FET, day-3, number of embryos not specified	Daily intravaginal progesterone gel 8% and oral dydrogesterone 40 mg until 8th-10th gestational week	Oral estradiol 8 mg and estradiol gel daily before cycle day 5
Zhang et al. [23] (5000 UI) China	(R). 1562.42 ± 560.91 (I) 1599.65 ± 505.66	(R)8.55 ± 2.40 (I) 8.53 ± 2.13	5000 UI of urinary hCG	5000 UI of urinary hCG + 0.1 mg of triptorelin	When the leading follicle reached 18 mm in diameter	FET, day-3; SET or DET,	200 mg of micronized progesterone twice daily intravaginal	Oral ethinylstradiol 75 mcg daily from cycle day 3 onwards and oral estradiol/dydrogesterone 8 mg daily once the endometrial lining thickness was > 8 mm
Zhang et al. [23] (10,000 UI) China	(R)1547.31 ± 557.14 (I) 1601.44 ± 503.26	(R)8.55 ± 2.42 (I). 8.60 ± 2.14	10,000 UI of urinary hCG	10,000 UI of urinary hCG of triptorelin	When the leading follicle reached 18 mm in diameter	FET, day-3; SET or DET	200 mg of micronized progesterone twice daily intravaginal	Oral ethinylstradiol 75 mcg daily from cycle day 3 onwards and oral estradiol/dydrogesterone 8 mg daily once the endometrial lining thickness was > 8 mm
[24] Iran	(R)1942 ± 691 (I) 1672 ± 597	(R)14.2 ± 0.24 (I). 13 ± 0.30	6500 IU of urinary hCG	6500 IU of urinary hCG + 0.2 mg of triptorelin	When at least three follicles reached ≥ 18 mm in diameter	ET, day-2; SET or DET	400 mg of micronized progesterone twice daily intravaginal	No
De Oliveira et al. [25] Brasil	NA	NA	6500 UI of recombinant hCG	6500 UI of recombinant hCG + 0.2 mg of triptorelin	NA	ET (not available)	1200 mg of micronized progesterone daily	No
Ozer et al. [19] Turkey	(R)2400.14 ± 1290.21 (I) 2385.91 ± 1290.80	NA	6500 UI of recombinant hCG	6500 UI of recombinant hCG + 0.2 mg of triptorelin	When the diameter of the precursor follicle reached 18–20 mm	ET, day-5 number of embryos not specified	90 mg vaginal progesterone gel 8% twice daily until the 12th week of gestation	No

R, single trigger; I, double trigger; R1, single trigger with coriofollitropin alpha; R2, single trigger without coriofollitropin alpha; I1, dual trigger with coriofollitropin alpha; I2, dual trigger without coriofollitropin alpha

### The number of metaphase II oocytes

#### Bologna criteria

Six studies assessed the number of metaphase II oocytes according to the Bologna criteria. The pooled effect size showed that dual triggering was associated with a significantly higher number of mature oocytes compared with hCG-only triggering (MD 0.68, 95% CI 0.30–1.05, *p* = 0.0004).

In subgroup analyses, this effect was statistically significant in non-randomized studies (MD 0.81, 95% CI 0.36–1.25, *p* = 0.0004), whereas a similar but non-significant trend was observed in randomized controlled trials (MD 0.36, 95% CI –0.42–1.13, *p* = 0.37).

**Fig. 2:**
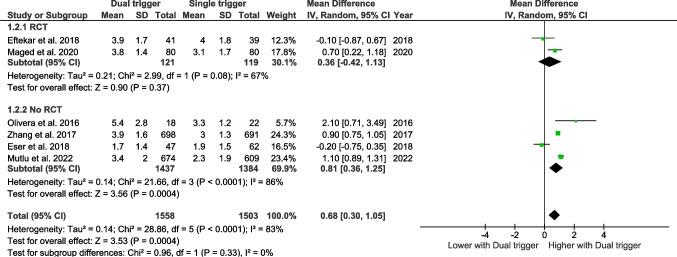
Forest plot of mature oocytes comparing dual trigger vs hCG trigger (Bologna studies)

No significant interaction was found between study design and treatment effect (test for subgroup differences *p* = 0.33), indicating that the magnitude of the effect did not differ significantly between randomized and non-randomized studies (Fig. 2).

#### Poseidon criteria

**Fig. 3:**
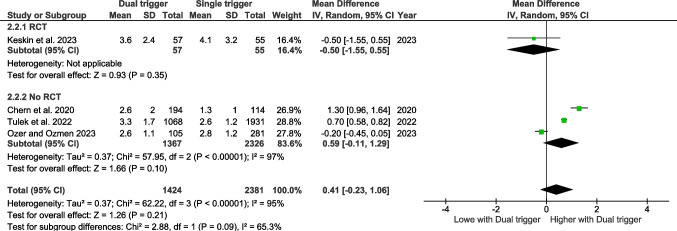
Forest plot of mature oocytes comparing dual trigger vs hCG trigger (POSEIDON studies)

Four studies have assessed the number of metaphase II oocytes retrieved according to the Poseidon criteria (Fig. 3). The pooled effect size does not reveal any significant effect.

### The number of oocytes retrieved

#### Bologna criteria

**Fig. 4:**
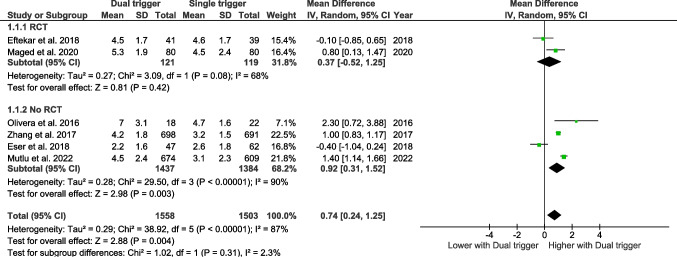
Forest plot of retrieved oocytes comparing dual trigger vs hCG trigger (Bologna studies)

Six studies have assessed the number of oocytes retrieved according to the Bologna criteria. The pooled effect size indicates that the number of oocytes retrieved is significantly higher in women who underwent the dual trigger than in the control group (WMD 0.74, CI 95% 0.24–1.25, *p* = 0.004) (Fig. 4).

#### Poseidon criteria

**Fig. 5:**
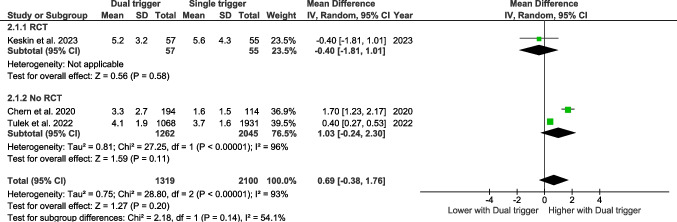
Forest plot of retrieved oocytes comparing dual trigger vs hCG trigger (POSEIDON studies)

Three studies have assessed the number of oocytes retrieved according to the Poseidon criteria. The pooled effect size does not reveal any significant effect (Fig. 5).

### Miscarriage rate

#### Bologna criteria

**Fig. 6:**
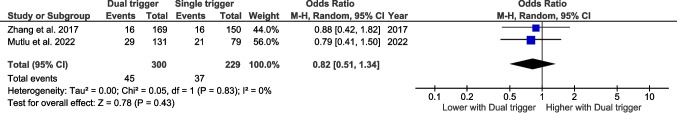
Forest plot of miscarriage rate comparing dual trigger vs hCG trigger (Bologna studies)

Two studies have assessed miscarriage rate according to the Bologna criteria. The pooled effect size does not reveal any significant effect (Fig. 6).

#### Poseidon criteria

**Fig. 7:**
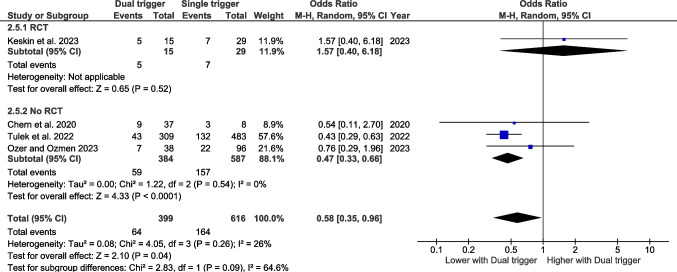
Forest plot of miscarriage rate comparing dual trigger vs hCG trigger (POSEIDON studies)

Four studies have assessed miscarriage rate according to Poseidon criteria. The pooled effect size indicates that conventional triggering is associated with a significantly lower miscarriage rate than the dual triggering approach (OR 0.58 CI 95% 0.35–0.96, *p* = 0.04) (Fig. 7).

### Clinical pregnancy rate

#### Bologna criteria

**Fig. 8:**
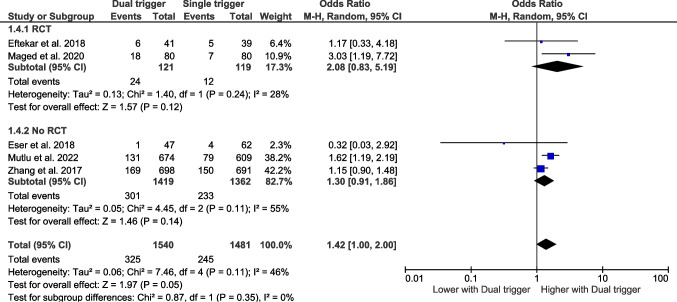
Forest plot of clinical pregnancy rate comparing dual trigger vs hCG trigger (Bologna studies)

Five studies have assessed the clinical pregnancy rates according to the Bologna criteria. The pooled effect size indicates that the dual trigger does not affect the clinical pregnancy rate (Fig. 8).

#### Poseidon criteria

**Fig. 9:**
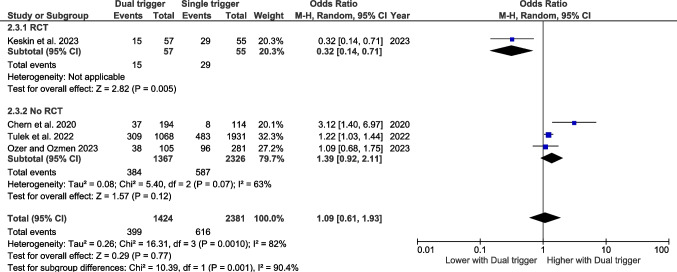
Forest plot of clinical pregnancy rate comparing dual trigger vs hCG trigger (POSEIDON studies)

Four studies have assessed the clinical pregnancy rate according to the Poseidon criteria. The pooled effect size indicates that dual trigger does not affect the clinical pregnancy rate (Fig. 9).

#### Risk of bias assessment

Risk of bias within studies is illustrated in Supplemental Table 1. Visual inspection of the funnel plots suggested some asymmetry; however, quantitative assessment using Egger’s regression test did not demonstrate statistically significant evidence of publication bias (*p* = 0.74). In addition, Begg’s test showed consistent results (*p* = 0.78). (Supplemental Fig. 1).

#### Sensitivity analysis

A sensitivity analysis excluding the non-randomized study at serious risk of bias [25] did not change the direction of the association, confirming the robustness of the primary results (Supplemental Fig. 2).

## Discussion

This review aimed to evaluate the potential benefits of dual triggering versus hCG-only triggering in poor responders undergoing controlled ovarian stimulation with a GnRH antagonist protocol and selected according to the Bologna or POSEIDON criteria.

Our analysis indicates that, among patients classified according to the Bologna criteria, dual triggering is associated with a higher number of retrieved and mature oocytes. However, this advantage was not confirmed in patients classified according to the POSEIDON criteria.

It is reasonable to hypothesize that the conflicting results observed in our study could be attributed, at least in part, to the persistent heterogeneity in the definition of poor responders, despite the seemingly strict Bologna and POSEIDON inclusion criteria.

As highlighted by Papathanasiou [27], five different phenotypes of poor ovarian response (POR) can be derived from various combinations of the Bologna criteria: (I) one previous poor response and age ≥ 40 years; (II) one previous poor response and abnormal ovarian reserve markers; (III) age ≥ 40 years and abnormal markers (the so-called "expected poor response"); (IV) a previous poor response in women aged ≥ 40 years with abnormal markers; and (V) two previous POR cycles after maximal stimulation. Thus, grouping such heterogeneous patients under a single label may introduce some degree of bias when comparing different treatment strategies, as this intrinsic clinical heterogeneity can limit the generalizability of pooled results and potentially mask differential responses to triggering strategies among specific subgroups.

Similarly, despite the improvements introduced by the POSEIDON criteria, recent critical analyses have revealed inconsistencies and incomplete reporting in studies that use these criteria.

This led to the development of the POSORT guidelines (POSEIDON Statement Of Reporting Trials) [28], a 20-item checklist aimed at improving reporting and ensuring more homogeneous patient characterization. POSORT recommends specifying which ovarian reserve marker (AFC, AMH, or both) is used and detailing the methods for their assessment to promote valid and reproducible research.

An important confounding factor in our analysis is patient age. Even within groups classified under the same criteria, age distributions varied widely. For instance, even when restricting the analysis to patients meeting the Bologna criteria, age distributions varied notably between studies: Eftekhar et al. reported a median age of 32–33 years, whereas Maged et al. [20] included women with a median age of 38 years. Similarly, within POSEIDON-based population, Chern et al. [18] focused on a cohort with a median age of 40 years (POSEIDON group 4), whereas Ozer et al. [19] examined a younger population, with a median age of 32 years. Interestingly, older patients appeared to benefit more from dual triggering [18, 20, 21] while younger patients [19, 22, 24] showed no significant advantage.These findings suggest that age-related oocyte competence may influence the effectiveness of dual triggering. A subgroup analysis of POSEIDON groups 3 (< 35 years) and 4 (≥ 35 years) would have been informative, but only Tulek et al. stratified patients accordingly. Such an analysis was therefore not possible and further research with extensive, well-designed studies is needed.

In this meta-analysis, the number of mature (MII) oocytes was selected as primary outcome. This parameter, together with the number of retrieved oocytes, represent the most direct and biologically coherent measure of trigger efficacy. These upstream outcomes allow minimizing the confounding influence of downstream factors such as sperm quality, embryo competence, laboratory performance, embryo-selection policies, luteal-phase support, and endometrial receptivity.

This approach aligns with the Vienna consensus [29], which identifies MII and oocyte yeld rate as key performance indicators of ovarian response.

Notably, the only previous meta-analysis on this topic, conducted by Sloth et al. [11], primarily focused on implantation, clinical pregnancy, and live birth rates, without evaluating oocyte yield as a primary efficacy endpoint. By prioritizing oocyte yield, our analysis provides a more proximal and biologically sound assessment of the impact of dual triggering.

From a clinical perspective, it is nevertheless essential to acknowledge the translational gap between increased oocyte yield and improved live birth rates.In particular, an increase in oocyte number does not automatically translate into a higher probability of live birth.

However, in poor responders, even modest improvements in oocyte yield may have clinically meaningful implications. In poor responders, where the reproductive margin is extremely narrow, maximizing the number of mature oocytes retrieved remains pivotal, as the retrieval of even a single additional oocyte may substantially influence the probability of achieving a live birth. Robust evidence from large population-based cohorts supports this concept: for istance, Oudendijk et al. [33] reported a pregnancy probability of only 0–7% with one retrieved oocyte, compared with 11.5–18.6% when four oocytes were obtained. Similarly, Sunkara et al. [34], analyzing more than 400,000 ART cycles, showed that even a single additional retrieved oocyte was associated with a significant increase in live birth rates among low responders.

Consistent with these observations, in our Bologna-defined subgroup, patients receiving dual triggering retrieved approximately one additional oocyte compared with those receiving a single trigger. Specifically, the mean number of MII oocytes was 3.6 ± 1.84 in the dual-trigger group versus 2.7 ± 1.66 in the single-trigger group, while the total number of retrieved oocytes was 4.3 ± 2.15 versus 3.3 ± 1.97, respectively. These findings provide biological and clinical plausibility to the observed benefit of dual triggering in this specific population.

To ensure the completeness of our analysis, clinical pregnancy and miscarriage rates were also assessed, although considered to be influenced by variables unrelated to the trigger intervention, particularly in poor responder populations.

When considering outcomes such as clinical pregnancy rates, defined by the Bologna criteria continued to show a significant benefit with double triggering. In contrast, the overall POSEIDON population did not appear to derive a benefit from dual triggering. It should be noted, however, that one of the included studies — the randomized controlled trial by Keskin et al. [16]—compared two populations with a notable age imbalance, with a mean age of 36.75 years in the double-trigger group versus 33.91 years in the single-trigger group. As embryo transfers were performed without preimplantation genetic testing for aneuploidy (PGT-A), this age difference may represent a substantial source of bias.

Regarding miscarriage rates, the only study supporting a benefit of double triggering was that by Tulek et al. [17], which demonstrated a reduced rate in both POSEIDON group 3 and group 4. The authors attributed this finding to the positive effects of GnRH agonists on both endometrial receptivity and embryo quality, supported by previous evidence of increased HOXA gene expression and improved embryonic development. Although these findings suggest possible biological mechanisms, they remain preliminary and cannot be generalized.

Regarding miscarriage rates, the only study reporting a benefit of dual triggering was that by Tulek et al. [17], which showed a reduced rate in both POSEIDON groups 3 and 4. The authors attributed this finding to potential biological effects of GnRH agonists on endometrial receptivity and embryo quality, supported by evidence of increased HOXA gene expression and improved embryonic development.However, this result appears to be largely driven by a single large retrospective study contributing most of the statistical weight, while the remaining studies did not demonstrate a consistent effect. Moreover, despite relatively standardized luteal-phase support, significant differences in embryo transfer practices were observed between groups, with a higher proportion of day-5 transfers and good-quality embryos in the dual-trigger arm.

These factors may confound the observed association making it difficult to determine whether the observed reduction reflects a true biological effect of dual triggering on embryo competence or is primarily driven by embryo selection and center-specific clinical practices.. Therefore, this finding should be considered exploratory and warrants confirmation in prospective studies with standardized post-retrieval management.

The strength of our article lies in the subgroup analysis conducted according to the Bologna and POSEIDON criteria, which aims to capture potential differences among distinct poor-responder populations. This strategy was intended to minimize heterogeneity and better identify clinical scenarios in which dual triggering could offer a real advantage. Nonetheless, some limitations must be acknowledged. The currently available literature on poor responders is heterogeneous, particularly with regard to study design and control of confounding—an established challenge in this field. We were fully aware of these constraints and addressed them by applying a rigorous methodological approach, including ROB-I/ROB-II assessments, a random-effects model, and evaluation of publication bias. Notably, despite the methodological variability, the direction and magnitude of the effect were consistent across studies, supporting the robustness of our findings.

In addition, only 10 studies were eligible for inclusion, and most were retrospective cohort studies, which are inherently limited by potential confounding factors. Such designs may introduce selection bias due to non-random patient inclusion, as well as information bias arising from incomplete or inconsistently recorded data. Unmeasured confounding related to ovarian reserve, stimulation protocols, and laboratory practices is also possible, as is selective reporting of key outcomes. The lack of blinding is another concern, as it may influence subjective outcomes, notably the assessment of embryo quality. In addition, measurement bias cannot be excluded, as most included studies—except that of Maged et al. [20]—did not specify the AMH assay used, potentially leading to misclassification of ovarian reserve.

Because one included study [25] was rated as having serious confounding bias, we performed a sensitivity analysis excluding it from the meta-analysis of oocyte yield. The pooled estimate remained essentially unchanged, supporting the stability of our findings despite the methodological limitations of some primary studies (Supplemental Fig. 2).

Beyond these intrinsic limitations of retrospective designs, additional methodological concerns may further reduce the strength of our conclusions. Selection bias warrants particular attention: although the Bologna and POSEIDON criteria are widely accepted, they may still cluster women with distinct prognostic profiles—such as differences in age or previous ovarian response. Without refined subgroup stratification, cross-study comparisons may therefore become misleading, potentially diluting any actual benefit of dual triggering in narrowly defined subsets of poor responders.

Although all studies followed the same COH regimen and the standard GnRH antagonist protocol, the type and dosage of gonadotrophins varied across studies, and it remains unclear whether such differences may have influenced oocyte-related outcomes. Nonetheless, available evidence suggests that moderate variations in hCG or GnRH-agonist dosage, as well as differences between recombinant and urinary hCG preparations, are unlikely to substantially affect oocyte maturation or related outcomes [30–32].

Additional heterogeneity arises from variations in luteal-phase support and embryo-transfer strategies (single versus double embryo transfer, day-3 versus day-5 transfers), as well as from incomplete reporting of PGT-A procedures. These factors call for caution when interpreting downstream reproductive outcomes such as clinical pregnancy and miscarriage rates.

This study underscores the critical need for precise characterization of poor responders in future trials. Stratification based on patient age, ovarian reserve markers, and standardized diagnostic criteria is essential to generate reliable and clinically meaningful evidence.

## Conclusion

Several interventions and protocols have been proposed to improve outcome in PORs, which include variations in the regimen for pituitary suppression and ovarian stimulation as well as the use of adjuvant therapies.

The limited and conflicting results regarding the potential benefits of dual triggering as an alternative to the standard single hCG triggering may be due to the limits of the Bologna and POSEIDON criteria as initially proposed.

These criteria have demonstrated high acceptability and overall good accuracy in identifying women with a poor reproductive prognosis. However, when applied to comparative analyses across different groups, their intrinsic heterogeneity may introduce some limitations. Overall, these observations suggest that the clinical effect of dual triggering is unlikely to be uniform across all poor responders and should be interpreted within the context of more specific patient phenotypes.

Future research should move beyond generic comparisons and focus on well-designed randomized controlled trials enrolling homogeneous subgroups of poor responders, such as specific POSEIDON categories, to assess the efficacy of different trigger strategies with the aim of generating clear and conclusive evidence.

To improve reproducibility and comparability, upcoming trials should explicitly adhere to POSORT reporting guidelines and adopt standardized dual-trigger regimens.

Moreover, future studies should report a core outcome set encompassing not only oocyte yield and maturity, but also live birth rate per initiated cycle and luteal-phase support requirements. This approach will enable a more comprehensive and clinically meaningful evaluation of trigger strategies in poor responders.

## Supplementary Information

Below is the link to the electronic supplementary material.

ESM 1 Supplementary Material 1 (PDF 148 KB)

ESM 2 Supplementary Material 2 (PDF 734 KB)

ESM 3 Supplementary Material 3 (DOCX 19.1 KB)

## Data Availability

The data that support the findings of this study are available from the corresponding author, upon reasonable request.
