# Correction: Efficacy of dual triggering in poor ovarian responders defined according to Bologna and POSEIDON criteria: a systematic review with meta-analysis

**DOI:** 10.1007/s10815-026-03845-x

**Published:** 2026-03-21

**Authors:** Antonio Mercorio, Alessandro Conforti, Nicola Pluchino, Panagiotis Drakopoulos, Matteo Giudice, Pierluigi Giampaolino, Alexandre Vallee, Vehbi Yavuz Tokgoz, Carlo Alviggi, Jean Marc Ayoubi

**Affiliations:** 1 Assisted Reproductive Technology Unit, Department of Gynecology, Hôpital Foch, Suresnes, France; 2 Department of Public Health, University of Naples Federico II, Naples, Italy; 3 Department of Neurosciences, University of Naples Federico II, Naples, Italy; 4 Division of Gynecology, Department Woman-Mother-Child, Centre Hospitalier Universitaire Vaudois (CHUV), University Hospital of Lausanne, Lausanne, Switzerland; 5 Institute of Life, IVF Unit, Athens, Greece; 6 Faculty of Medicine, European University Cyprus, Nicosia, Cyprus; 7 Centre for Reproductive Medicine, Universitair Ziekenhuis Brussel, Vrije Universiteit Brussel, Brussels, Belgium; 8 Department of Obstetrics and Gynaecology, University of Alexandria, Alexandria, 21526 Egypt; 9 Department of Epidemiology-Data-Biostatistics, Delegation of Clinical Research and Innovation (DRCI), Foch Hospital, Suresnes, 92150 France; 10 Reproductive Endocrinology and Infertility Unit, Department of Obstetrics and Gynecology, School of Medicine, Eskişehir Osmangazi University, Eskişehir, Turkey

**Correction: Journal of Assisted Reproduction and Genetics**

10.1007/s10815-026-03821-5

The original online version of this article was revised: The fourth author's name was incorrectly spelled as "Panagiotis Drakopulos." It should be "Panagiotis Drakopoulos". The original article has been corrected.

