# Supplementary figures and images for: Efficacy of dual triggering in poor ovarian responders defined according to Bologna and POSEIDON criteria: a systematic review with meta-analysis
Source: J Assist Reprod Genet. 2026 Feb 6;43(4):1063–77. doi: 10.1007/s10815-026-03821-5 (PMC13103112; doi:10.1007/s10815-026-03821-5)

Supplemental figure 1 Funner plot, Egger test and trim and fill method

S  
T  
A  
N  
D  
A  
R  
D  
  
E  
R  
R  
O  
R

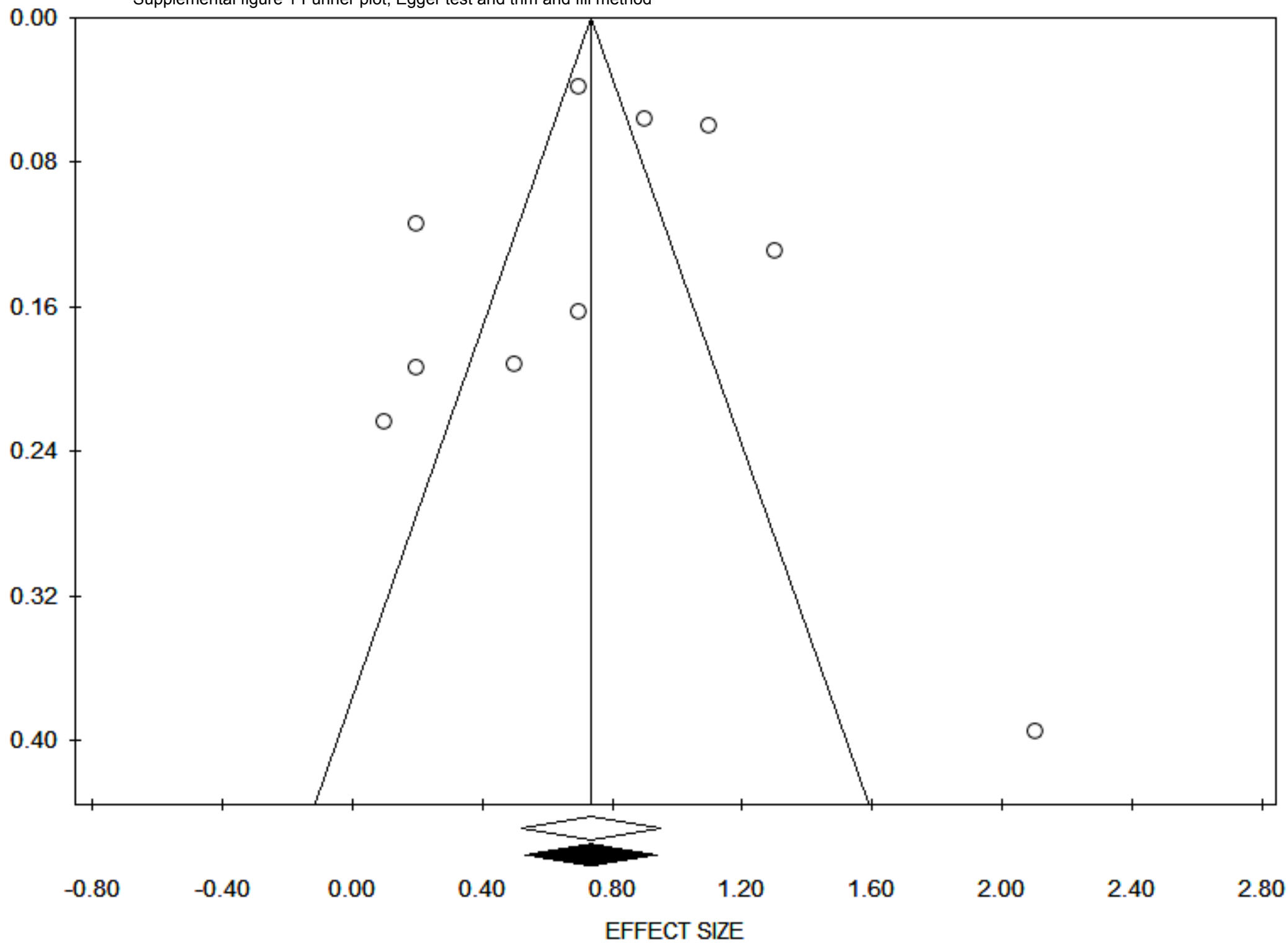

Supplement: Supplementary file 1 — Supplementary Material 1 (PDF 148 KB) [file 10815_2026_3821_MOESM1_ESM.pdf]
