# Supplementary material for: Efficacy of dual triggering in poor ovarian responders defined according to Bologna and POSEIDON criteria: a systematic review with meta-analysis
Source: J Assist Reprod Genet. 2026 Feb 6;43(4):1063–77. doi: 10.1007/s10815-026-03821-5 (PMC13103112; doi:10.1007/s10815-026-03821-5)

Sensitivity analysis for the primary outcome (number of mature oocytes) excluding the non-randomized study by De Oliveira et al. (2016).

Study or Subgroup	Dual trigger Mean	SD	Total	Single trigger Mean	SD	Total	Weight	Std. Mean Difference IV, Random, 95% CI	Year
**1.1.1 RCT**									
Eftekar et al. 2018	3.9	1.7	41	4	1.8	39	13.0%	-0.06 [-0.50, 0.38]	2018
Maged et al. 2020	3.8	1.4	80	3.1	1.7	80	17.8%	0.45 [0.13, 0.76]	2020
**Subtotal (95% CI)**			**121**			**119**	**30.8%**	**0.22 [-0.27, 0.71]**	
Heterogeneity: Tau² = 0.09; Chi² = 3.36, df = 1 (P = 0.07); I² = 70%									
Test for overall effect: Z = 0.88 (P = 0.38)									
**1.1.2 No RCT**									
Olivera et al. 2016	5.4	2.8	18	3.3	1.2	22	0.0%	0.99 [0.33, 1.66]	2016
Zhang et al. 2017	3.9	1.6	698	3	1.3	691	27.1%	0.62 [0.51, 0.72]	2017
Eser et al. 2018	1.7	1.4	47	1.9	1.5	62	15.1%	-0.14 [-0.52, 0.24]	2018
Mutlu et al. 2022	3.4	2	674	2.3	1.9	609	27.0%	0.56 [0.45, 0.67]	2022
**Subtotal (95% CI)**			**1419**			**1362**	**69.2%**	**0.44 [0.20, 0.68]**	
Heterogeneity: Tau² = 0.03; Chi² = 14.00, df = 2 (P = 0.0009); I² = 86%									
Test for overall effect: Z = 3.65 (P = 0.0003)									
**Total (95% CI)**			**1540**			**1481**	**100.0%**	**0.37 [0.16, 0.58]**	
Heterogeneity: Tau² = 0.04; Chi² = 21.75, df = 4 (P = 0.0002); I² = 82%									
Test for overall effect: Z = 3.42 (P = 0.0006)									
Test for subgroup differences: Chi² = 0.63, df = 1 (P = 0.43), I² = 0%									

Std. Mean Difference
IV, Random, 95% CI

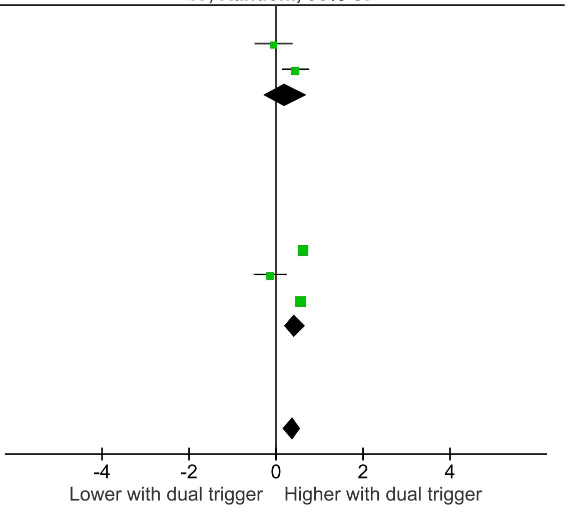

Supplement: Supplementary file 2 — Supplementary Material 2 (PDF 734 KB) [file 10815_2026_3821_MOESM2_ESM.pdf]
