# Supplementary material for: Efficacy of dual triggering in poor ovarian responders defined according to Bologna and POSEIDON criteria: a systematic review with meta-analysis
Source: J Assist Reprod Genet. 2026 Feb 6;43(4):1063–77. doi: 10.1007/s10815-026-03821-5 (PMC13103112; doi:10.1007/s10815-026-03821-5)
Supplement: Supplementary file 3 — Supplementary Material 3 (DOCX 19.1 KB) [file 10815_2026_3821_MOESM3_ESM.docx]

| **Supplemental Table. Risk of bias in non-randomized studies according to Rob I tool** | | | | | | | |
| --- | --- | --- | --- | --- | --- | --- | --- |
| **Study*** | **Confounding** | **Selection** | **Measurement classification of Intervention** | **Deviations from intended intervention** | **Missing data** | **Measurements of outcomes** | **Reported results** |
| Chern et al. 2020 | Low | Serious | Low | Low | Low | Low | Low |
| De Oliveira et al. 2016 | Serious | Serious | Low | Low | Serious | Low | Serious |
| Eser et al. 2018 | Moderate | Low | Low | Low | Moderate | Low | Moderate |
| Mutlu et al. 2022 | Low | Low | Low | Low | Low | Low | Low |
| Ozer at al. 2023 | Low | Low | Low | Low | Moderate | Low | Low |
| Tulek et al. 2022 | Low | Low | Low | Low | Low | Low | Low |
| Zhang et al. 2017 | Low | Low | Low | Low | Low | Low | Low |

| **Supplemented Table. Risk of bias randomized studies** | | | | | | | |
| --- | --- | --- | --- | --- | --- | --- | --- |
| **Study*** | **Random sequence generation (selection bias)** | **Allocation concealment (selection bias)** | **Blinding of participants and personnel (performance bias)** | **Blinding of outcome assessment (detection bias)** | **Incomplete outcome data (attrition bias)** | **Selective reporting (reporting bias)** | **Other bias** |
| Keskin et al. 2023 | Low | Low | Unclear | Low | Unclear | Unclear | Low |
| Maged et al. 2020 | Low | Unclear | High | High | Low | Unclear | Unclear |
| Eftekar et al. 2018 | Moderate | Low | Low | Low | Moderate | Low | Moderate |
